# Deforestation and Forest Fragmentation in South Ecuador since the 1970s – Losing a Hotspot of Biodiversity

**DOI:** 10.1371/journal.pone.0133701

**Published:** 2015-09-02

**Authors:** María Fernanda Tapia-Armijos, Jürgen Homeier, Carlos Iván Espinosa, Christoph Leuschner, Marcelino de la Cruz

**Affiliations:** 1 Sección de Ecología y Sistemática, Departamento de Ciencias Naturales, Universidad Técnica Particular de Loja, Loja, Ecuador; 2 Plant Ecology, Albrecht von Haller Institute for Plant Sciences, University of Göttingen, Göttingen, Germany; 3 Área de Biodiversidad y Conservación, Departamento de Biología y Geología, ESCET, Universidad Rey Juan Carlos, Madrid, Spain; Instituto de Pesquisas Ecológicas, BRAZIL

## Abstract

Deforestation and fragmentation are major components of global change; both are contributing to the rapid loss of tropical forest area with important implications for ecosystem functioning and biodiversity conservation. The forests of South Ecuador are a biological ‘hotspot’ due to their high diversity and endemism levels. We examined the deforestation and fragmentation patterns in this area of high conservation value using aerial photographs and Aster satellite scenes. The registered annual deforestation rates of 0.75% (1976–1989) and 2.86% (1989–2008) for two consecutive survey periods, the decreasing mean patch size and the increasing isolation of the forest fragments show that the area is under severe threat. Approximately 46% of South Ecuador’s original forest cover had been converted by 2008 into pastures and other anthropogenic land cover types. We found that deforestation is more intense at lower elevations (premontane evergreen forest and shrubland) and that the deforestation front currently moves in upslope direction. Improved awareness of the spatial extent, dynamics and patterns of deforestation and forest fragmentation is urgently needed in biologically diverse areas like South Ecuador.

## Introduction

Deforestation and forest fragmentation are among the main components of global change [1] and both contribute to the rapid loss of tropical forest area with important implications for ecosystem functioning and biodiversity conservation [2, 3]. Deforestation has been directly linked to species extinctions [4], loss of ecosystem services [5], enhanced emission of CO2 and other greenhouse gases [6, 7], and changes in the structure and habitat quality of aquatic ecosystems [8].

Deforestation not only reduces forest area but also changes the landscape configuration [9]. Fragmentation increases habitat isolation and edge effects and reduces the size of forest patches [10]. The resulting smaller patches exist under different abiotic conditions and meta-population environments than the un-fragmented forest [11], thereby limiting the available resources needed to maintain local populations, presenting barriers that some species are unable to cross, and influencing species interactions [12, 13].

According to the Food and Agriculture Organization of the United Nations (FAO) [14], Ecuador has maintained the highest deforestation rates of South America during the last 20 years (annual rates of 1.5% and 1.8% for the 1990–2000 and 2001–2010 periods, respectively). As in other tropical countries, agricultural expansion, wood extraction for fuel, commercial logging, the establishment of oil palm, cacao and banana plantations, bioethanol cropping, mining and road construction are main drivers of ongoing land cover changes [15–17].

In Ecuador, the highest deforestation rates reported to date have been detected in the northwestern Amazon and the northwestern coastal regions (e.g. [18–22]). With the exception of the studies of Keating [23] and Thies et al. [24], there is only scarce information on deforestation rates in South Ecuador, which is thought to be an important front of deforestation in the country. At the same time, this region is of particular interest and value for biodiversity conservation [25].

South Ecuador (the provinces of Loja and Zamora Chinchipe) has been identified as a center of biodiversity (e.g. [26, 27]) and is to a large part situated within the Tropical Andes biodiversity hotspot, which is considered as the richest hotspot on earth [28, 29]. Furthermore, the south-western part of Loja province is part of the Tumbes – Chocó – Magdalena biodiversity hotspot which includes the unique dry forests of Ecuador and Peru [30].

South Ecuador is characterized by a very specific flora which differs markedly from the rest of the country and has a high degree of endemism [31, 32]. According to Valencia [33], from the 4,011 endemic species of Ecuador, 639 are registered for Loja and 568 for Zamora Chinchipe and of these 515 are exclusive for the region. Podocarpus National Park, the most important protected forest area in the region, has the highest number of endemic vascular plants species (211) of all other protected areas in the country [33]. Its high biodiversity and endemism were the reason to include a great share of the region in the recently created Podocarpus—El Cóndor Biosphere Reserve [34].

Recent studies focusing on small areas in this region reported high rates of deforestation [24, 35]. Drastic effects of deforestation and fragmentation on species richness and composition have been documented (e.g. [5, 27, 36, 37]). Against this background, this study aimed at describing land cover change and changes in forest spatial configuration in the highly diverse South Ecuadorian forest region since the 1970`s by 1) determining deforestation rates in the region during the periods 1976–1989 and 1989–2008, 2) identifying which are the natural forest types that have suffered the highest conversion rates and 3) evaluating the changes in the spatial patterns of forest cover over time by means of selected landscape metrics.

## Methods

### Study Area

**Fig 1:**
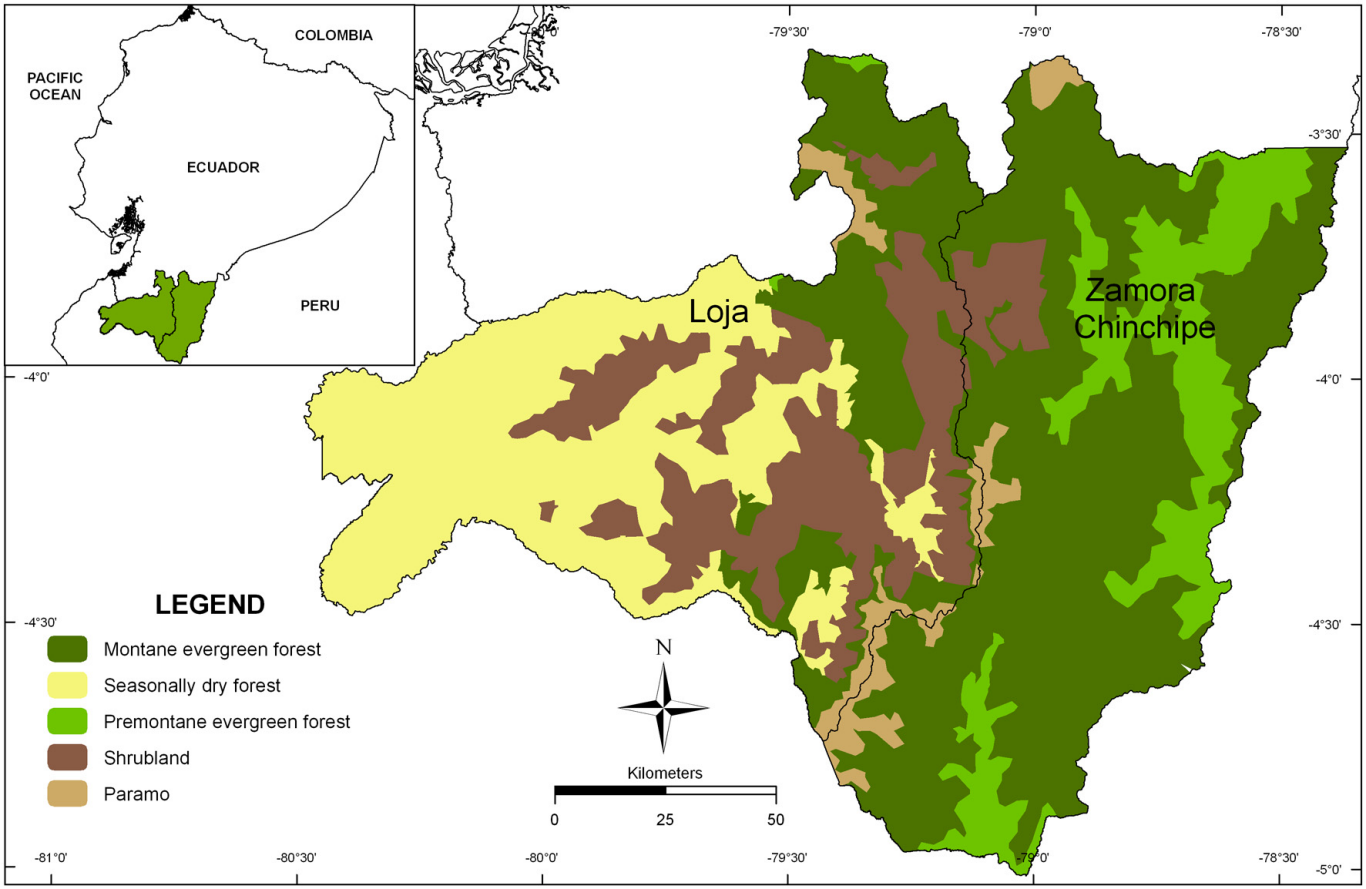
Location of the Study area. Distribution of the main natural vegetation categories in the Loja and Zamora Chinchipe Provinces in South Ecuador.

Loja and Zamora Chinchipe provinces are located between 78° and 80°W and 3° and 5°S and cover approximately 21,631 km^2^ in South Ecuador (Fig 1). Both provinces are geographically separated by the Cordillera Real, the eastern range of the Ecuadorian Andes. This region is a topographically diverse area where elevation ranges from 105 to 3,866 m a.s.l. [38]. The thermal gradient ranges from 7°C to 25°C mean annual temperature. It depends not only on elevation but also on the exposition of the macro- and meso-relief with respect to the prevailing wind direction [39, 40]. The precipitation regime is determined by the Andean ridge; the eastern Andean slopes are moist in contrast to the (semi-) arid climate of the western side of the range [41, 42]. The precipitation ranges from 500 mm to 8,000 mm per year; some inter-mountain dry areas receive less than 500 mm of annual precipitation [42, 43]. Soil conditions are highly variable, depending on elevation, bedrock and climate (e.g. [44]).

### Geographic data

Historic land cover patterns for 1976 and 1989 were obtained from black and white aerial photographs (scale 1:60,000) because the availability of historic satellite images in general and of cloud-free satellite scenes in particular is limited for the study area. A total of 486 aerial photographs were used to obtain the land cover mosaic for the first study year (1976) and 469 aerial photographs for the second study year (1989). The aerial photographs were provided by the National Mapping Agency (IGM) from the government projects Carta Nacional 1976–1978 and Carta Nacional 1986–1989.

The land cover map for the third study year (2008) was derived from Advanced Spaceborne Thermal Emission and Reflection Radiometer Data (ASTER, 15 m resolution) scenes type 1B. A total of 17 scenes (60x60 km) were acquired from the USGS GloVis portal. To obtain a complete scenes mosaic with a cloud cover of less than 20%, we used scenes from three consecutive years (2006–2008), because especially the eastern part of the study area is covered with clouds most of the year [23, 45].

For pre-processing and land cover classification of aerial photographs and satellite images (described below), we used maps of roads and rivers derived from 55 topographic maps [46], a 90-m digital elevation model [38] and all available land use maps of smaller areas within our study region from different periods [23, 35, 47].

### Land-cover classification

Patches of natural forests existing in 1976 and 1989 were drawn from aerial photographs with the use of a stereoscope. To distinguish between the three categories natural cover areas, other cover types (non-natural covers) and cloud-covered areas, we used a visual interpretation using color, texture and context criteria [48]. All drawn polygons were scanned, digitized and individually geo-referenced. A minimum of 15 control points were used to reference each aerial image, using well defined permanent objects such as rivers and road intersections. Polygons that were not consistent with the mosaic were redrawn. The interpreted land cover mosaics of 1976 and 1989 were transferred as a vector map to ArcGIS (9.2) [49].

Land cover maps from 2008 for the Loja and Zamora Chinchipe provinces [50, 51] were derived from Aster satellite images, using the first three bands. ASTER scenes were acquired with an initial radiometric and geometric calibration [52]. In mountainous areas, it is necessary to integrate ancillary data [53, 54]. Thus, the Aster scenes were ortho-rectified with a DEM (90 m) and the river network using a second-order polynomial model [53, 55]. Atmospheric correction was applied to all scenes using the Cost Model [56] which incorporates deep object subtraction, Rayleigh dispersion and a procedure that calculates the absorption effects by atmospheric gasses. The topographic correction was made using the IDRISI SELVA hillshade control procedure [57] with a DEM (SRTM 90 m) to remove differences in solar illumination influenced by relief, one of the principal problems arising in the analysis of satellite scenes in mountainous areas with rugged topography [58, 59].

A total of 630 (non-randomly distributed) ground control points were recorded in order to conduct a supervised classification to distinguish the “natural cover” areas from non natural cover areas called “other covers” (which include crops, pastures, plantations, degraded forest and urban areas). The maximum likelihood criterion was used to assist in the classification of overlapping signatures, in which pixels were assigned to the class of highest probability [60]. The selection of the ground-truthing points was limited by site accessibility that depended on available roads and topography.

In order to facilitate the discrimination of difficult covers, both historical aerial photographs and Aster scenes were classified following specific criteria which considered the characteristics of the studied landscape in each period, the criteria are detailed below:

#### Pastures

In Ecuador natural grasslands are characteristic of paramo vegetation and specifically for South Ecuador they are restricted to areas above 2800 m a.s.l. [19]. We assumed that pastures below 2800 m a.s.l. in the study area are the result of human activities and thus included them under “non-natural covers”. Paramo usually presents a continuous surface (S1a Fig) while pastures exhibit highly parceled patterns (S1b Fig). Additionally, pastures were distinguished from paramo by the generally brighter grey tones in the aerial photographs (S1c Fig).

#### Tree plantations

In the eastern part of the study area there are still no forest plantations, in the western part there are plantations of pines (mostly *Pinus patula)* and eucalypts (*Eucalyptus spp*.). In the aerial photographs tree plantations show lower densities (S2a Fig) and a lighter tone compared with natural forests and uniform spatial pattern as consequence of the equally spaced trees within rows (S2b Fig).

#### Secondary and degraded forests

In this study, we do not discriminate between primary and secondary forest (it is not possible using aerial photographs), we were more interested in discriminate the forest without human intervention from the forest with human intervention. Thus, we used forest density to classify forest as natural covers or other covers (S3a Fig). Dense forest was classified as natural cover (S3b Fig) and those forests with some grade of intervention (e.g. areas of forest with gaps resulting from selective logging) were considered within class other covers (non natural—covers) (S3a Fig).

### Accuracy assessment

The visual interpretation of the land cover maps of 1976 and 1989 was checked by a person with excellent knowledge of the covered localities and validated against other thematic maps of smaller areas [23, 35, 47]. The validation of historic land cover at ground truthing points was impossible due to the changes registered in the landscape since the photos were taken.

To validate the precision with which land cover map of 2008 discriminated natural cover areas from other covers (non-natural covers) a new set of 541 testing ground truthing points was used. We constructed a confusion matrix to obtain the precision of the classification comparing the class identified for each sample point with the cover derived from ASTER scenes classification [48]. The resulted accuracy assessment was given by the values obtained in the global accuracy, producer´s accuracy, and user´s accuracy measures and the kappa index Eq (1) where π_0_ is and observational probability of agreement and πe is a hypothetical expected probability of agreement under an appropriate of baseline constraints such as total independence of observer classifications [61].

$$\kappa =\frac{\pi_{0}-\pi_{e}}{1-\pi_{e}}$$ (1)

### Deforestation at regional level and for the different forest types

In contrast to other studies (e.g. [62]) we only quantify the loss of original forest area and disregard areas of regenerating or secondary forests. Our experience is that deforested areas in the study area do not attain the structural attributes or species composition of undisturbed forests even after some decades of natural succession [5].

Deforestation analyses were conducted at two levels. At regional level we calculated the annual deforestation rates for each period (1976–1989; 1989–2008) using the differences in natural cover area. In addition, we calculated the annual deforestation rates for the principal natural forest types in the region.

We used the spatial information of the vegetation classification map for Ecuador proposed by Sierra et al. [19]. And then simplified the categories according to the classifications proposed by Balslev and Øllgaard [63] and by Homeier et al. [64] (Table 1).

**Table 1:** Description of the natural vegetation categories used in this study, combining the vegetation classifications proposed for South Ecuador by Balslev & Øllgaard [62], Homeier et al. [63] and Sierra [19].

CATEGORY (Balslev & Øllgaard [62], Homeier et al. [63])	DESCRIPTION	CORRESPONDENT CATEGORY (Sierra [19])
Premontane evergreen forest (PEF)	Species—rich forest growing from 500 m to 1 300 m a.s.l. being characteristic for the eastern escarpment of the Andes. Maximum tree height is 30–40 m.	1. Amazon foothill evergreen forest, 2. Coastal foothill evergreen forest
Montane evergreen forest (MEF)	Forest growing from 1 300 m to 3 100 m a.s.l. being characterized by a high diversity and abundance of epiphytes. The trees reach up to 30 m in the lowermost areas and to less than 10 m in the highest areas. These forests are mainly located on the slopes of the Cordillera Real.	3. Western Andes upper montane evergreen forest, 4. Eastern Andes upper montane evergreen forest, 5. Amazon cordillera lower montane evergreen forest, 6. Western Andes lower montane evergreen forest, 7. Southeastern Andes lower montane evergreen forest, 8. Amazon cordillera montane evergreen forest, 9. Western Andes montane cloud forest, 10. Eastern Andes montane cloud forest
Paramo (PA)	Contains two types of paramo: herbaceous and shrub paramo. Both are found above 2 800 m a.s.l. The shrub paramo is a natural cover unique for South Ecuador.	11. Herbaceous paramo, 12. Dry paramo, 13. Southern Andes shrub páramo
Shrubland (SL)	Vegetation characteristic of interandean valleys between 1 200 and 3 000 m a.s.l. and the western slope of the Andes below 1 800 m a.s.l. This category includes dry and semi-dry shrublands which differ in density and composition of species.	14. Southern Andes montane humid shrub, 15. Southern Andes montane dry shrub
Seasonally dry forest (SDF)	Located in the western part of the study area, where annual precipitation ranges from 500 to 2 500 mm with a long period of drought. This category includes dry deciduous and semi-deciduous forests which all grow below 1 000 m a.s.l.	16. Coastal foothill deciduous forest, 17. Coastal lowland deciduous forest, 18. Western Andes lower montane semi deciduous forest, 19. Coastal foothill semi deciduous forest, 20. Coastal lowland semi deciduous forest

Both analyses excluded the surface covered by paramo since this vegetation type is not dominated by trees. To obtain deforestation rates, we used the compound-interest-rate formula Eq (2) that was proposed by Puyravaud [65] and used in similar studies [60, 66], where A1 and A2 are the area cover by natural forest at time t1 and t2, respectively and P is the annual deforestation rate.

$$P=\frac{100}{t_{2}-t_{1}}\text{ln}\frac{A_{2}}{A_{1}}$$ (2)

The presence of areas with clouds and shadows in both aerial photographs and Aster scenes produced areas without information. A mask was generated with all the areas without information from any of the three periods. This mask was extracted from each period in order to keep the analyzed area comparable.

### Change detection analyses

A post classification change-detection methodology was used for investigating to what land cover type the natural forest areas were transformed. This approximation used the thematic maps obtained after land cover classification to implement a comparison pixel by pixel between two periods maps [67, 68]. For this analysis, first we calculate how much surface of the natural cover in 1976 was converted to pastures, crops, degraded forest, plantations and urban areas in 2008, using as a first scene the land cover map of 1976 and as the final scene the categories representing non-natural covers in the land cover map of 2008. Second, we calculate how much area of the different vegetation types was converted to the same non-natural covers used above (pastures, crops, degraded forest, plantations and urban areas) but in this case we use as a first scene the reclassified map that shown the area covered by the different vegetation types in 1976.

### Fragmentation analysis

A set of key landscape metrics was used to quantify and compare the spatial configuration of native forest fragments, taking into account that the selected metrics did not include redundant information [69]. We used the program FRAGSTATS 3.4 [70] to calculate the following parameters: (1) the number of fragments of natural forest (PN: Patch number); (2) area of each individual patch (PA: Patch area); (3) the percentage of the landscape occupied by the largest fragment of natural forest (LPI: Largest patch index); (4) the mean size of natural forest patches (MPS: mean patch size); (5) the number of patches per 100 ha (PD: Patch density; (6) the degree of isolation of natural forest patches resulting of measure the ratio between the size and proximity of all patches whose edges are within 1 km of the focal patch (MPI: Mean proximity index); (7) the total patch size remaining after removing a specific buffer edge (TCA: Total core area); 8) the complexity of patch shapes compared to a standard shape (MSI: Mean shape index) and 9) the sum of the lengths of all edge segments in the landscape (TE: Total edge length).

## Results

### Accuracy assessment

For the validation of the 2008 land cover map we used a confusion matrix (Table 2). According to Foody et al. [71] the overall accuracy shows the percentage of cases correctly allocated. Our results showed an overall accuracy of 92.4%, which means that the 2008 land cover classification had a high performance to discriminate the pixels with natural cover from those with other covers (non-natural covers). 94.5% of the areas classified as natural cover were really natural cover and 90.3% of the areas classified as other covers were really other covers.

**Table 2:** Confusion matrix obtained from the accuracy assessment of the 2008 land cover map of South Ecuador.

	REFERENCE				
CLASSIFIED	Natural Cover	Other Covers	Total	User´s accuracy	Commission error
**Natural Cover**	257	15	272	94.49	5.51
**Other Covers**	26	243	269	90.33	9.67
**Total**	283	258	**541**		
**Producer´s accuracy**	90.81	94.19			
**Omission error**	9.19	5.81			
**Overall accuracy**	**0.92**				

According to Congalton [72] the Kappa index could be considered as a powerful technique to provide accuracy information derived from a confusion matrix. For this study, the Kappa index was 0.84 which means that the land cover classification for 2008 could be considered as almost perfect according to the parameters proposed by Landis and Koch [61].

### Deforestation and land cover change patterns

**Fig 2:**
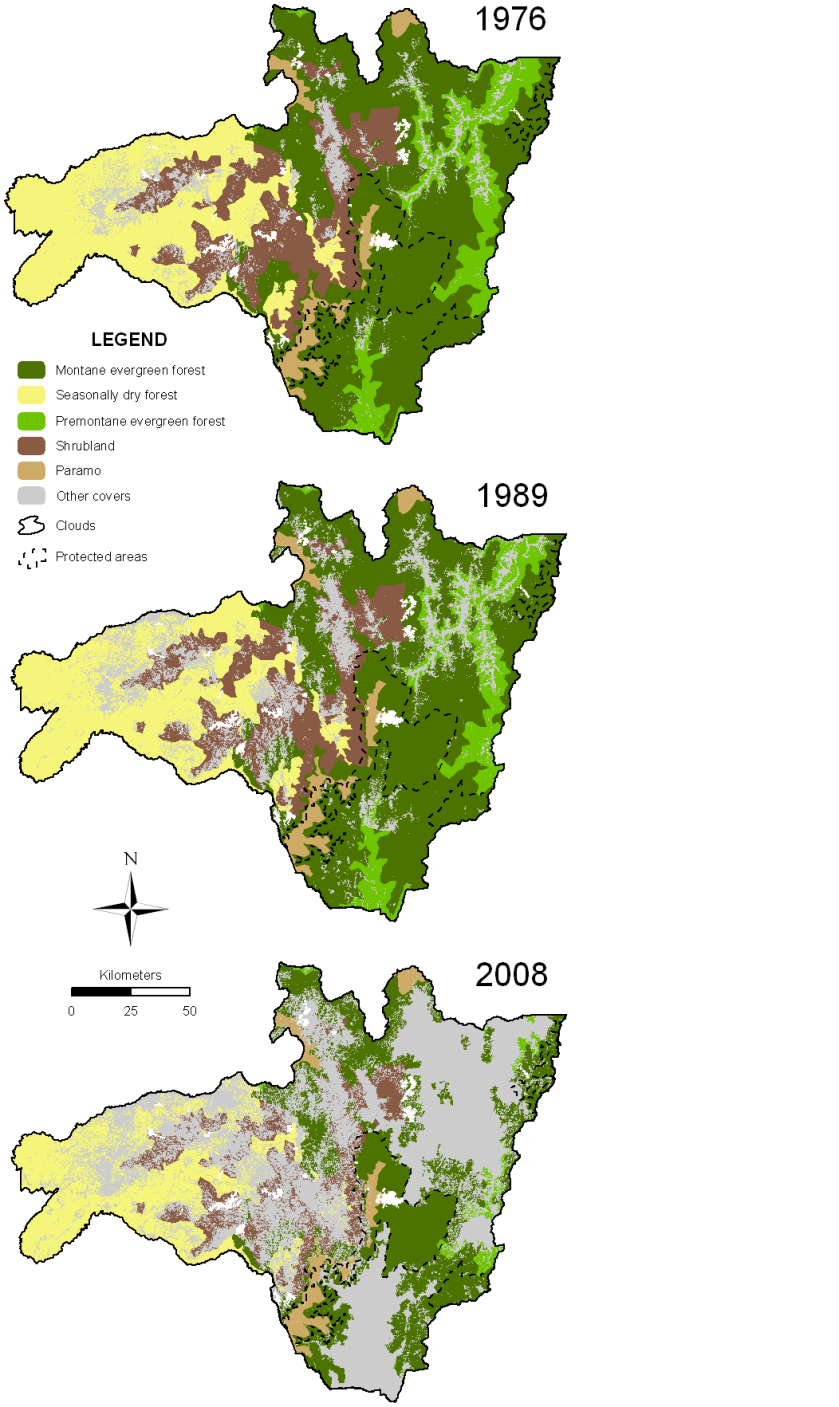
Land cover maps for the years 1976, 1989, 2008. Maps display the spatial distribution patterns of the different land cover types in South Ecuador for the three studied years. The black dashed polygons show the boundaries of the protected areas that belong to the national system of protected areas (PANE).

**Fig 3:**
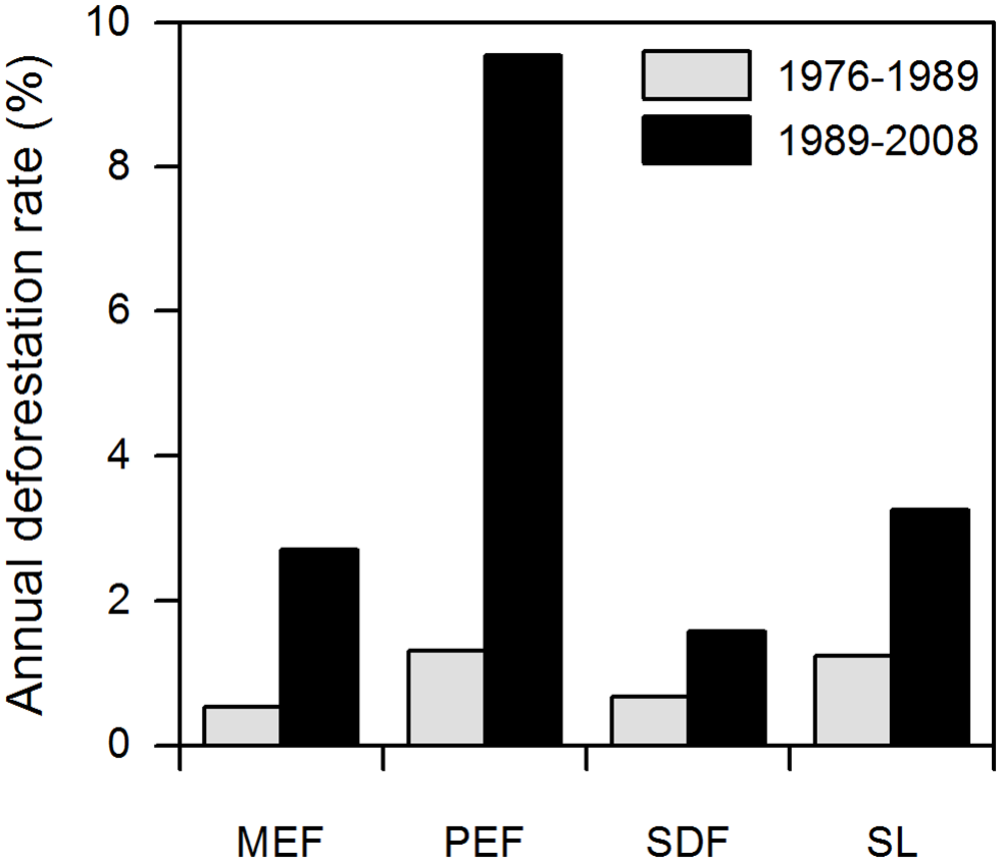
Annual deforestation rates for different vegetation types in South Ecuador. Annual deforestation rates in four natural vegetation types in the study area for the periods 1976–1989 and 1989–2008. MEF = Montane evergreen forest, PEF = Premontane evergreen forest, SDF = Seasonally dry forest, SL = Shrubland.

Changes in land cover (Table 3) were derived from the land cover maps of 1976, 1989 and 2008 (Fig 2). The area covered by original vegetation decreased during this time by approximately 46%, from 19,500 km2 in 1976 to 10,550 km2 in 2008. The annual deforestation rate in South Ecuador’s forest area for the period from 1976–1989 was 0.75%; it increased considerably to 2.86% in the 1989–2008 period. The average deforestation rate for the entire 32-yr-long study period was 2.01%. Premontane evergreen forest and shrubland were the vegetation types that suffered the highest conversion rate during the whole study period (Fig 3, Table 3).

**Table 3:** Area covered by different natural forest types, other covers (non-natural covers) and clouds in 1976, 1989 and 2008 in South Ecuador.

YEAR	1976		1989		2008	
COVER TYPE	(km^2^)	(%)	(km^2^)	(%)	(km^2^)	%
Premontane evergreen forest (PEF)	2 033.58	9	1 714.99	8	279.3	1
Montane evergreen forest (MEF)	9 221.08	43	8 605.03	40	5 150.71	24
Paramo (PA)	714.83	3	710.72	3	662.83	3
Shrubland (SL)	2 966.51	14	2 526.06	12	1 361.86	6
Seasonally dry forest (SDF)	4 563.93	21	4 183.76	19	3 097.50	14
Clouds (CL)	272.82	1	272.82	1	272.82	1
Other covers (OC)	1 858.25	9	3 617.62	17	10 805.98	50
**Total**	**21 631**	**100**	**21 631**	**100**	**21631**	**100**

During the 32 years of the study period, 3,954 km2 of natural forest have been converted to degraded forest, a similar area (3,654 km2) has been converted to pastures, and another 631 km2 to crop lands. The change detection analysis (Table 4) shows that the premontane evergreen forest and the montane evergreen forest were mainly transformed to degraded forest (51% and 27% of the initial area, respectively), and the shrubland and dry forest were mostly converted to pastures (33% and 18%, respectively). The only natural forest type with a relevant transformation to crops was dry forest (9%). Conversion of natural forest to plantations or urban areas was of minor importance.

**Table 4:** Changes of natural vegetation types of other covers in South Ecuador since 1976 to 2008.

Other Covers	Crops		Pastures		Plantations		Degraded Forests		Urban Areas	
Montane evergreen forest (MEF)	Total Converted Surface (km2)	%	Total Converted Surface (km2)	%	Total Converted Surface (km2)	%	Total Converted Surface (km2)	%	Total Converted Surface (km2)	%
**Premontane evergreen forest (PEF)**	97	1.1	1218	13.2	18	0.2	2444	26.5	5	0.1
**Seasonally dry forest (SDF)**	19	0.9	613	30.1	0	0.0	1041	51.2	3	0.1
**Shrubland (SL)**	439	9.6	832	18.2	1	0.0	87	1.9	10	0.2
**Paramo (PA)**	75	2.5	980	33.0	19	0.6	354	11.9	3	0.1
**Montane evergreen forest (MEF)**	1	0.1	11	1.6	3	0.4	28	3.9	0	0.0
**Total**	631	14.2	3653	96.2	41	1.2	3954	95.4	21	0.5

### Fragmentation patterns

**Fig 4:**
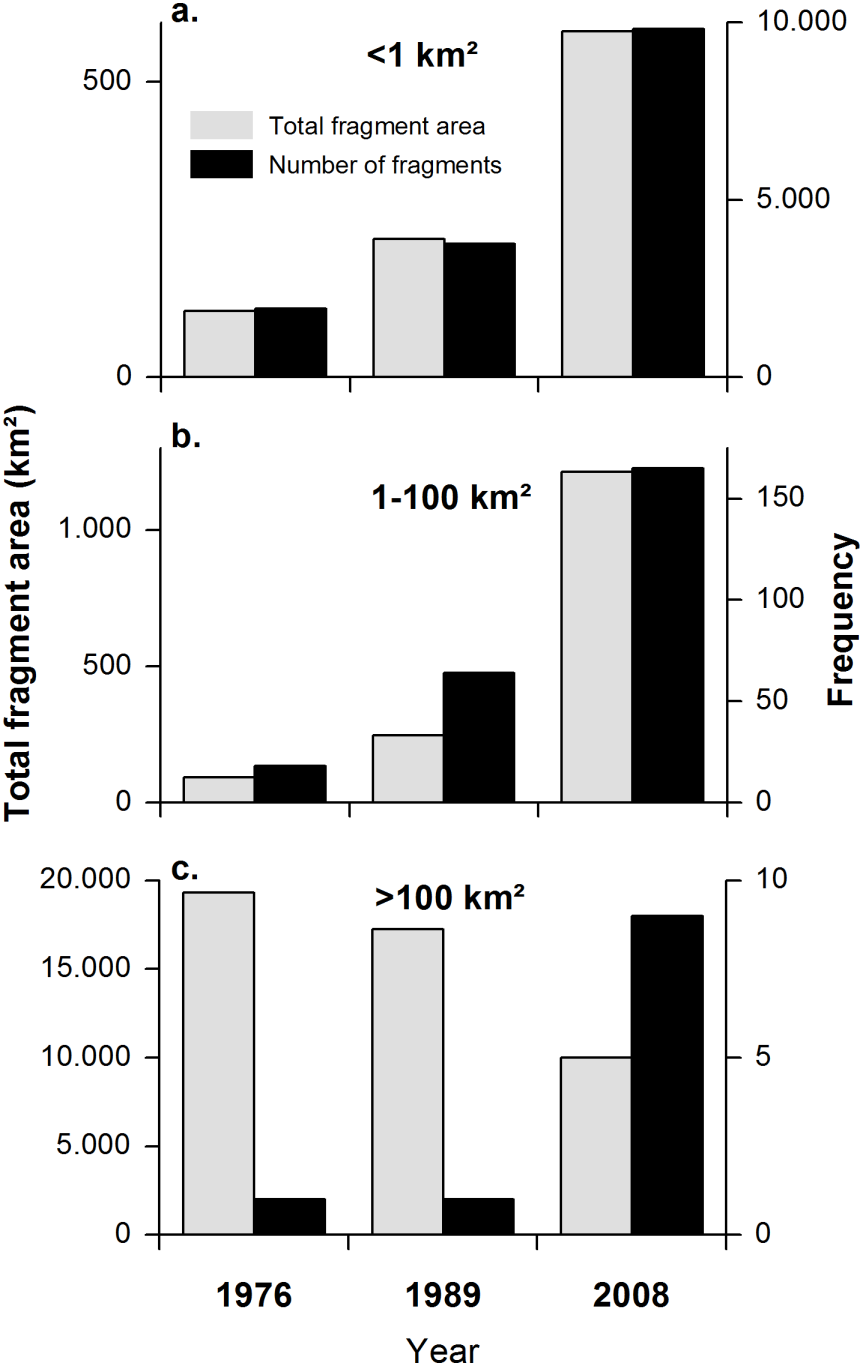
Variation of forest fragment size and total fragment area for 1976, 1989 and 2008. The figure shows the increase in the number of forest fragments (black bars) and the decreased in the cumulative area of the forest fragments (grey bars) of three different fragment size classes (<1km^2^, 1–100km^2^, >100km^2^) in each studied year.

The total number of forest patches increased from 1957 in 1976 to 3,831 in 1989, and to 9,988 in 2008 representing a 500% increase relative to the number of fragments present in 1976 (Fig 4). In 1976, the landscape contained one large continuous forest patch of 19,296 km2 that occupied 89% of the study area, while the remaining natural forest area was distributed to many small fragments of less than 1 km^2^ size. In 1989, the largest natural forest patch still occupied 80% of the landscape, but in 2008, this largest patch had been greatly reduced to not more than 19% of the study area. The remaining natural forest area is today concentrated in a few patches of more than 100 km^2^ size and a large number of small fragments with less than 1 km^2^. Mean forest fragment size decreased more than ten-fold from 15.1 km2 in 1976 to 1.4 km2 in 2008, which mostly results from the dissection and conversion of the initial large patch.

The process of fragmentation is further evident from the marked increase in patch density from 7 to 72 patches per 100 km^2^ (Table 5). The increasing total edge length (from 19,278 km to 51,109 km) and the growing mean shape index value (from 1.5 to 1.69) indicate increasing irregularity in the shape of natural forest patches and an increasing susceptibility to potential edge effects. The total core area of remaining natural forest considering a buffer zone of 300 m decreased by 70% from 1976 to 2008 to less than 5000 km^2^ today (Table 5). The mean core area per forest patch was reduced from 25 km^2^ (1976), to 12 km^2^ (1989) and just 3 km^2^ (2008).

**Table 5:** Changes in spatial configuration of natural forests in South Ecuador during the period from 1976 to 2008.

LANDSCAPE INDICES	1976	1989	2008
Total area of forest (km^2^)	19,500	17,741	10,550
Total number of patches	1,958	3,832	9,988
Mean patch size (km^2^)	15.1	6.3	1.4
Patch density (number of patches/100 km^2^)	7	16	72
Largest patch index (%)	89	80	19
Total edge length (km)	19,278	30,270	51,109
Mean shape index	1.5	1.59	1.69
Total forest core area^a^ (km²)	16,338	13,238	4,989
Mean proximity index	1,065,518	714,713	87,673

^a^ For the calculation of forest core area, we considered a buffer zone of 300 m width.

Another result of fragmentation is the increasing isolation of natural forest patches due to the replacement of natural forest by other land cover types. According to McGarigal [70] the mean proximity index, as a measure of the grade of isolation, decreased since the neighborhood is less occupied by natural forest patches. In this study this particularly occurred in the second period after 1989 (Table 5).

## Discussion

### Deforestation patterns

The natural forests of South Ecuador have suffered high conversion rates during the last thirty years with an intensification of this process during the last decade. During the first studied period (1976–1989), the annual deforestation rate (0.75%) was similar to the 0.70% decline of old-growth forest in Central Ecuador between 1963 and 1983 reported by Wunder [73]. Both rates are lower than the 1.17% reported from the North Ecuadorian Amazon for 1973 to 1985 [21].

The discovery of oil reserves in the Ecuadorian Amazon during the 1960’s led to the construction of new roads which accelerated the colonization of new areas in the lowlands. This was probably the first cause of forest loss in this zone [22]. In contrast, South Ecuador conserved large areas of natural forests until the 1980s, mainly in the eastern part that was then sparsely inhabited and with limited accessibility.

The Ecuadorian Agrarian Reform in 1964 promoted the colonization and clearance of previously forested areas to make them productive cropland or pastures nationwide [74]. However in the studied region the small size of existing settlements and the limited accessibility to natural areas delayed deforestation until recently.

We found that the annual deforestation rate increased considerably during the 1989–2008 period (2.86%), coinciding with Jokisch and Lair [75] who observed that at national level deforestation was accelerated during the 1990´s. During this period, the rate observed in this study was similar to that observed in the Northwest of the country (Lopez [76]: 2.2% in the Santiago and Cayapas rivers watersheds during 1993–2001 period), and half the rate observed in the Northeastern Amazon (Pan et al. [77]: 4.73% in Sucumbíos during the period 1986–1999). We assume that population growth combined with the expansion of the road system in South Ecuador (starting from the year 2000) increased the accessibility of until then unexplored areas and additionally made profitable the extraction of timber at lower cost [78].

In the study area, deforestation is principally concentrated in the bottoms of the valleys and lower slopes, with many small forest patches in varying states of degradation remaining within the most heavily impacted areas. Similar to other Latin American countries, the deforested areas are mainly used as pastures [79]. Our results show that the Andean pre-montane evergreen forest, the Seasonally dry forest and the Shrubland had been converted for large-scale cattle ranching documenting that this is not only a characteristic feature of the humid Amazon lowland in Brazil, Ecuador and Bolivia as reported by Geist and Lambin [80]. Andean pre-montane evergreen forest apparently has optimal thermal conditions for cattle ranching but due to the low soil fertility in the study area, many pastures are not very productive and therefore are frequently abandoned after a few years [5]. With regards to the seasonally dry forest, the establishment of pastures for cattle ranching seems to be the leading factor of tropical dry forest conversion [81, 82]. Kauffman et al. [83] estimated that the highest aboveground biomass losses in a Mexican dry forests occurred as a result of biomass burning, which is a common practice of peasants in order to convert dry forest to pastures. At the same time and in the short term, forest burning increases the pH and inorganic nitrogen of the soil, reducing the capacity to adapt of dry forest native species and increasing the vulnerability to alien species invasion [84].

In the eastern part of the study area the deforestation front seems to have moved upslope in the valleys through the different forest belts (e.g. the annual deforestation rate for the montane evergreen forest increased in the second survey period from 0.5% to 2.7% which is related to areas where the pre-montane evergreen forest had already been transformed before). In the western part, where seasonally dry forest and shrubland predominated there is not a clear frontline and the deforestation seems to result from diffuse smallholder activities. Precisely, these smallholders’ activities and the absence of large pasture or crop areas had produced a highly dynamic landscape where shrubland patches are scattered through the flat areas and the hill slopes. The shrubland showed the second highest deforestation rate in the study area. As shown by Schulz et al. [85] the deforestation process in shrubland is commonly characterized by the transition of shrubland to agricultural land followed by a later conversion of agricultural land to pasture or bare lands.

### Fragmentation patterns

In addition to the overall reduction of forest area, we found an increase in the number, isolation and irregularity of forest patches and a decrease in the size of patches reflecting the ongoing fragmentation of forest habitats. The intensification of forest fragmentation since 1989 seems to be related to the increase in accessibility that was mainly caused by the construction of new highways and rural roads. More fragmented woodlands (e.g. areas with more isolated, irregular and smaller fragments) usually occur near roads and rivers and towns and cities where the human population has increased considerably during the last decades. Hawbaker et al. [86] demonstrated a positive relationship between the increases in road density and the changes in landscape patterns (e.g. patch area and patch shape) and house density. In South Ecuador there are no specific studies that show this relation but Peters et al. [78] mentioned the decisive role that roads construction had on land reclamation in Ecuador and thereby on the changes observed in landscape patterns.

The factors related with the fragmentation process go beyond physical factors like road construction. Heterogeneity of socio-economic, demographic and other factors results in different types of changes in the landscape patterns. In the eastern part of South Ecuador, higher poverty and a shortage of adult labor result in the dominance of cattle ranching. Thus, the farms are characterized by large pasture areas around dwellings, small areas used for subsistence agriculture, and forest patches (< 20 km^2^) persist only in the most inaccessible areas of the farms. In contrast, Marquette [87] noted that in north-eastern Ecuador, where a combination of small—scale agricultural activity and cattle ranching predominates, approximately 80% of small farmers clear only small areas of forest. In Ecuadorian dry ecosystems the major remaining surface of forest is distributed to patches of more than 10 km^2^ that does not mean that fragmentation is low but rather than deforestation is the dominant process [88].

In the study area, the largest conserved forest patches persisted in 2008 in areas with a protection status such as national protected areas, private reserves, or communitarian protected forests, highlighting the importance of in situ conservation strategies. However, if the high deforestation rates are maintained and fragmentation is going on, the remaining forest will soon be reduced to isolated forest patches that cannot fully meet their conservation purpose [89] with a reduced ecological functionality and capacity to conserve species richness (e.g. [90, 91]). It reveals the importance to increase the protected area, especially in dry forest where conservation must be considered a prior task for Ecuador because of the high levels of endemism and the small extent of this natural vegetation type in the country [88, 92].

The progressive fragmentation in the study area may have serious consequences for local species with high requirements to their habitat (e.g. charismatic species such as *Tremarctos ornatus*, *Puma concolor* or *Tapirus pinchaque* which often require available habitat areas >2,000 km^2^) or for rare species with small population sizes and restricted geographical ranges (e.g. more than 1,000 endemic plant species are present in the area) [93]. Studies in other tropical hotspots showed that fragments of 1 km^2^ (approximately 9,000 fragments in our study area) lose one half of their species in <15 years (e.g. [94]) and that less than 50% of all midsized and large mammals persist in fragments <5 km^2^, even if the species are matrix—tolerant (e.g. [95]). Additionally, the loss of species results in locally impoverished and increasingly homogenized tree assemblages, where old-growth tree flora is replaced by a small subset of pioneer or successional tree species [96–98].

## Conclusions

Improved awareness of the spatial extent, dynamics and patterns of deforestation and forest fragmentation is urgently needed in biologically diverse areas like South Ecuador. Our study shows that this region, where only 9.8% of the surface area is under governmental protection, should be in the urgent focus of conservation initiatives, especially since new mining projects will soon open access to the larger forest tracts left in the southeastern part of the Zamora-Chinchipe province. Since there may be positive feedback between human land use, future climatic change [99] and increasing atmospheric nutrient deposition [100] the threats to most of the studied ecosystems are probably even larger than predicted from our land use trajectory.

## Supporting Information

S1 Fig Aerial photograph (IGM, 1976) that shows landscape mosaic of Saraguro in South Ecuador. Paramo (lighter tone) of Saraguro—Yacuambi wetland system dominated by herbaceous species limiting with forest (darker tone); b) Mosaic of pastures (lighter tone) and forest (darker tone), c) Agricultural zone around the town of Saraguro where a mosaic with high patchiness could be observed.
(TIFF)

S2 Fig Aerial photographs of Loja—Vilcabamba road. a) Aerial photographs (IGM, 1976) close to Loja in the Cajanuma sector that shows a younger plantation of *Pinus patula*, the grey tones of plantation areas, pastures and forests are different, b) Aerial photograph (2008) of the same plantation in the Cajanuma sector that shows the linear patterns that characterizes this cover. The aerial photograph in section b) was obtained and provided by the Ecuadorian Project SIGTIERRAS (Ministerio de Agricultura, Ganadería, Acuacultura y Pesca; Proyecto Sistema Nacional de Información y Gestión de Tierras Rurales e Infraestructura Tecnológica).
(TIFF)

S3 Fig Aerial photograph (IGM, 1989) of Rio Zamora basin. a) Dense forest (darker tone) with gaps that show human intervention (lighter tone), the yellow polygons delimit the dense forest area that was classified as natural cover. b) Continuous surface of dense forest.
(TIFF)
