# Correction: Deforestation and Forest Fragmentation in South Ecuador since the 1970s - Losing a Hotspot of Biodiversity

**DOI:** 10.1371/journal.pone.0142359

**Published:** 2015-11-03

**Authors:** María Fernanda Tapia-Armijos, Jürgen Homeier, Carlos Iván Espinosa, Christoph Leuschner, Marcelino de la Cruz

A heading is missing from the left-most column in Table 4. The first column should be labeled Natural vegetation types. Please see the corrected Table 4 here.

**Table 4:** Changes of natural vegetation types of other covers in South Ecuador since 1976 to 2008.

	Crops		Pastures		Plantations		Degraded Forests		Urban Areas	
**Natural vegetation types**	Total Converted Surface (km2)	%	Total Converted Surface (km2)	%	Total Converted Surface (km2)	%	Total Converted Surface (km2)	%	Total Converted Surface (km2)	%
**Montane evergreen forest (MEF)**	97	1.1	1218	13.2	18	0.2	2444	26.5	5	0.1
**Premontane evergreen forest (PEF)**	19	0.9	613	30.1	0	0.0	1041	51.2	3	0.1
**Seasonally dry forest (SDF)**	439	9.6	832	18.2	1	0.0	87	1.9	10	0.2
**Shrubland (SL)**	75	2.5	980	33.0	19	0.6	354	11.9	3	0.1
**Paramo (PA)**	1	0.1	11	1.6	3	0.4	28	3.9	0	0.0
**Total**	631	14.2	3653	96.2	41	1.2	3954	95.4	21	0.5
