# Supplementary figures and images for: Deforestation and Forest Fragmentation in South Ecuador since the 1970s – Losing a Hotspot of Biodiversity
Source: PLoS One. 2015 Sep 2;10(9):e0133701. doi: 10.1371/journal.pone.0133701 (PMC4557835; doi:10.1371/journal.pone.0133701)

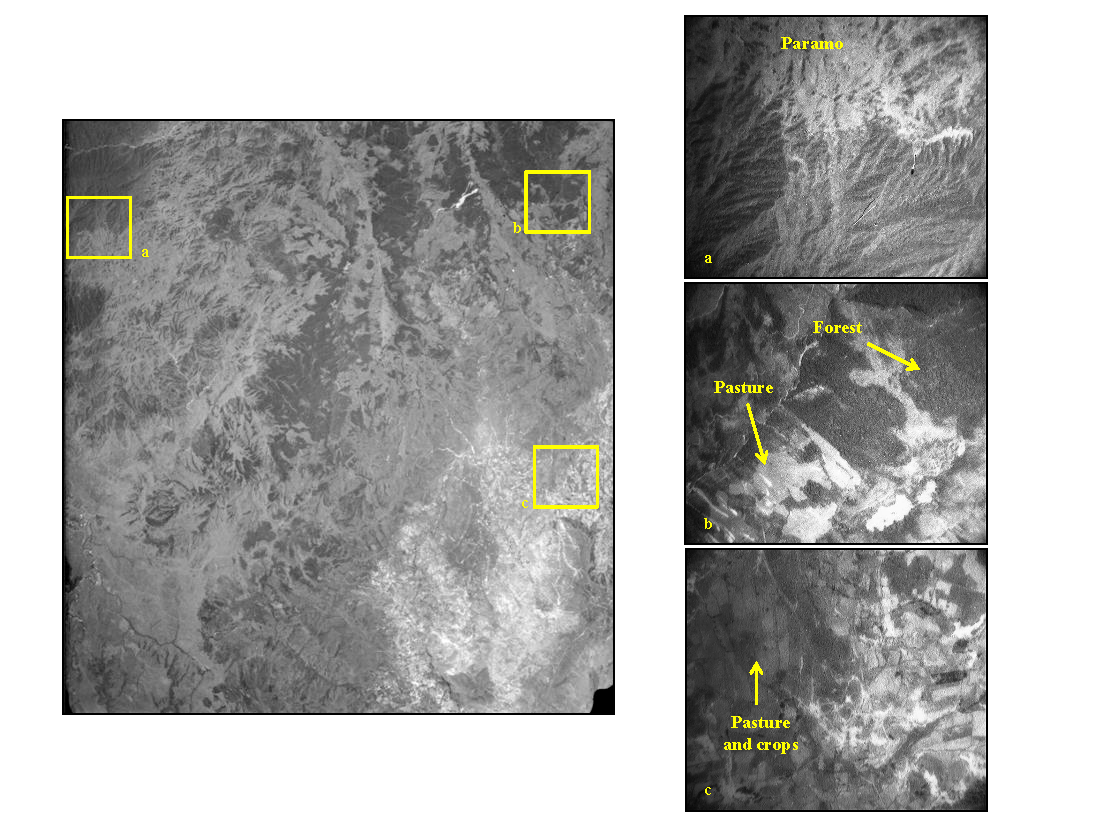

Supplement: S1 Fig — Paramo (lighter tone) of Saraguro—Yacuambi wetland system dominated by herbaceous species limiting with forest (darker tone); b) Mosaic of pastures (lighter tone) and forest (darker tone), c) Agricultural zone around the town of Saraguro where a mosaic with high patchiness could be observed. (TIFF) [file pone.0133701.s001.tiff]

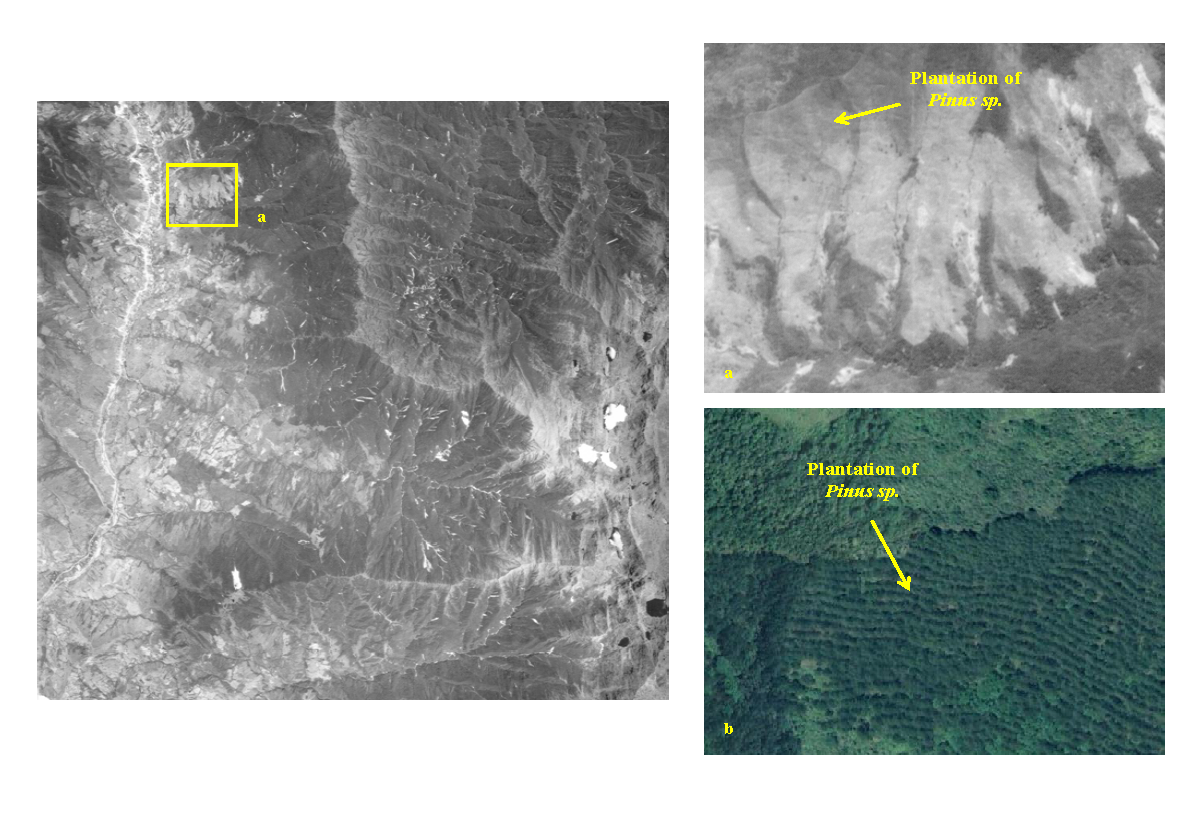

Supplement: S2 Fig — a) Aerial photographs (IGM, 1976) close to Loja in the Cajanuma sector that shows a younger plantation of Pinus patula, the grey tones of plantation areas, pastures and forests are different, b) Aerial photograph (2008) of the same plantation in the Cajanuma sector that shows the linear patterns that characterizes this cover. The aerial photograph in section b) was obtained and provided by the Ecuadorian Project SIGTIERRAS (Ministerio de Agricultura, Ganadería, Acuacultura y Pesca; Proyecto Sistema Nacional de Información y Gestión de Tierras Rurales e Infraestructura Tecnológica). (TIFF) [file pone.0133701.s002.tiff]

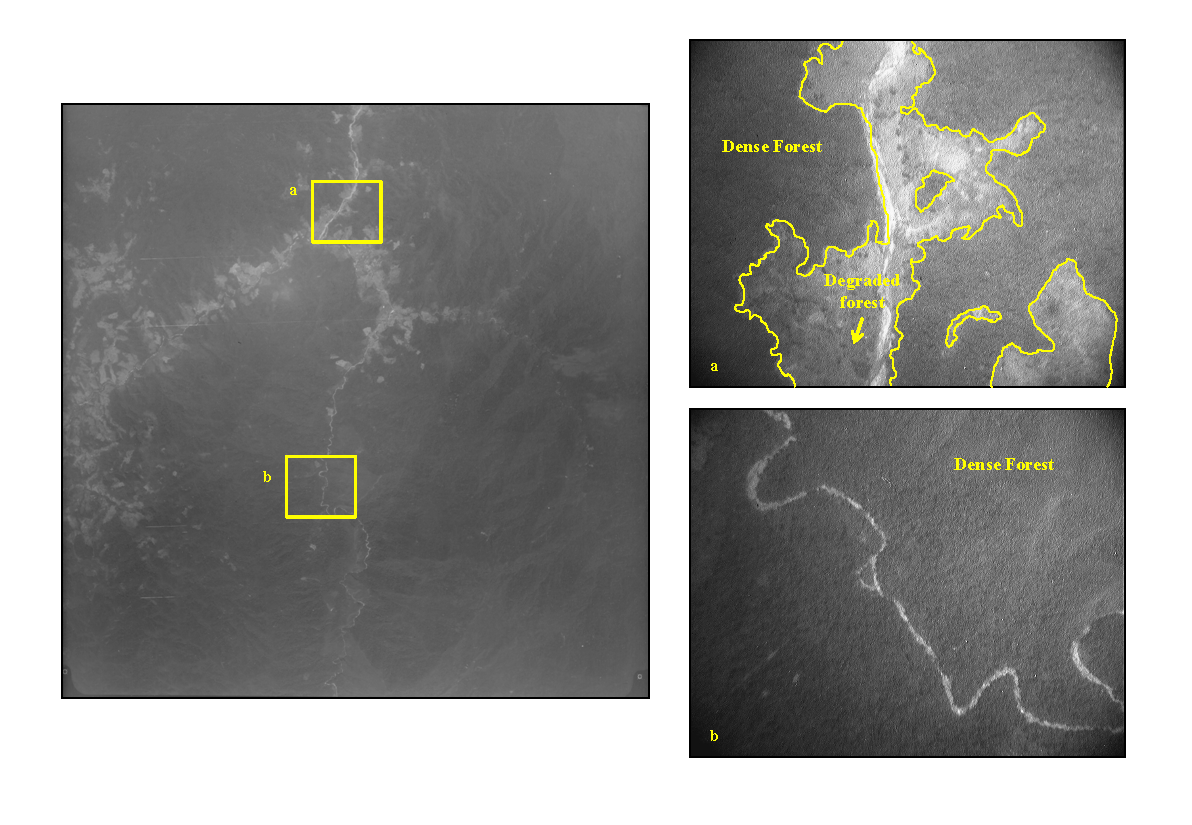

Supplement: S3 Fig — a) Dense forest (darker tone) with gaps that show human intervention (lighter tone), the yellow polygons delimit the dense forest area that was classified as natural cover. b) Continuous surface of dense forest. (TIFF) [file pone.0133701.s003.tiff]
